# Chronic invasive *Aspergillus Fumigatus* rhinosinusitis with intracranial extension

**DOI:** 10.1016/j.idcr.2026.e02618

**Published:** 2026-05-26

**Authors:** Tobias Kremsmayer, Nikita Rajbhandari, Reed Jaworski, Xiangyu Xie

**Affiliations:** a University of Illinois Chicago College of Medicine, Chicago, USA; b Department of Diagnostic Radiology, University of Illinois Chicago, Chicago, USA; c Department of Internal Medicine, University of Illinois Chicago, Chicago, USA

**Keywords:** Chronic invasive fungal disease with intracranial extension, Aspergillus fumigatus, Antifungal medication therapy, Type 2 diabetes mellitus

## Abstract

A 63-year-old male with type 2 diabetes mellitus (hemoglobin A1c: 8.2%) presented with progressively worsening blurry vision, headache, and unsteadiness. MRI of the brain and orbit revealed an infiltrating lesion at the cribriform plate with orbital and intracranial involvement. After extensive diagnostic evaluation including repeat biopsies, DNA analysis on biopsied tissue ultimately revealed *Aspergillus fumigatus*. During several multidisciplinary discussions with the patient and his family, surgical intervention was decided against due to associated morbidity and concern for adequate source control. Despite intensive antifungal medication management, including amphotericin, isavuconazole, and micafungin, for more than a year, there was disease progression with vision loss and brain abscess formation. This case highlights the diagnostic challenges contributing to the poor prognosis associated with chronic invasive fungal rhinosinusitis and the need for early evaluation and multidisciplinary intervention.

**Case Illustrated**

Chronic invasive fungal rhinosinusitis (CIFRS) is a rare disease most seen in older individuals with a history of diabetes mellitus (DM) or immunocompromise [1]. The insidious process of CIFRS along with the subacute presentation and symptom variability provides diagnostic challenges for providers [1]. CIFRS with intracranial spread particularly carries high mortality and morbidity [1], [2], [3]. Treating these patients surgically involves extensive resection, so invasive treatment is often avoided in lieu of systemic antifungal therapy with triazoles [1].

A 63-year-old male with a history of type 2 DM (hemoglobin A1c: 8.2%) initially presented for progressively worsening blurry vision, headache, and unsteadiness for several months. MRI of the brain and orbit revealed an infiltrating lesion at the cribriform plate with orbital and intracranial involvement ( Figure 1: A, Figure 2: A1). An aggressive pattern of bone destruction was also seen ( Figure 3). After extensive diagnostic evaluation including repeat biopsies, DNA analysis ultimately revealed *Aspergillus fumigatus*. At this time, the patient had lost all vision in the left eye. During several multidisciplinary discussions with the patient and his family, surgical intervention was decided against due to associated morbidity and concern for adequate source control. While combined surgical debridement plus systemic antifungals has been shown to improve outcomes in CIFRS when compared to medication treatment alone, overall mortality for intracranial aspergillosis remains high [4], [5]. The patient was discharged on oral isavuconazole and IV amphotericin.

**Fig. 1:**
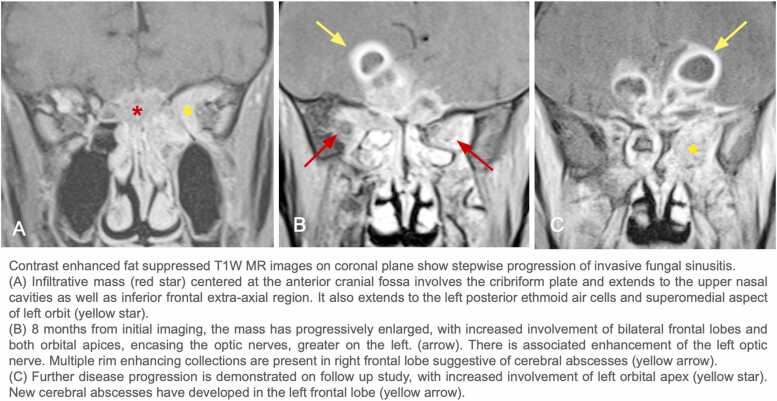
Contrast enhanced fat suppressed T1W MR images on coronal plane show stepwise progression of invasive fungal sinusitis.

**Fig. 2:**
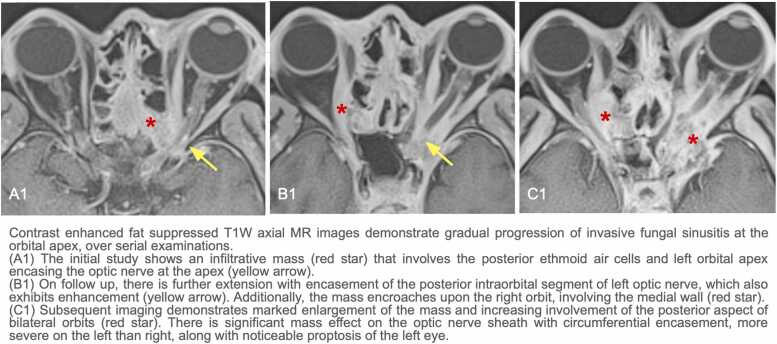
Contrast enhanced fat suppressed T1W axial MR images demonstrate gradual progression of invasive fungal sinusitis at the orbital apex, over serial examinations.

**Fig. 3:**
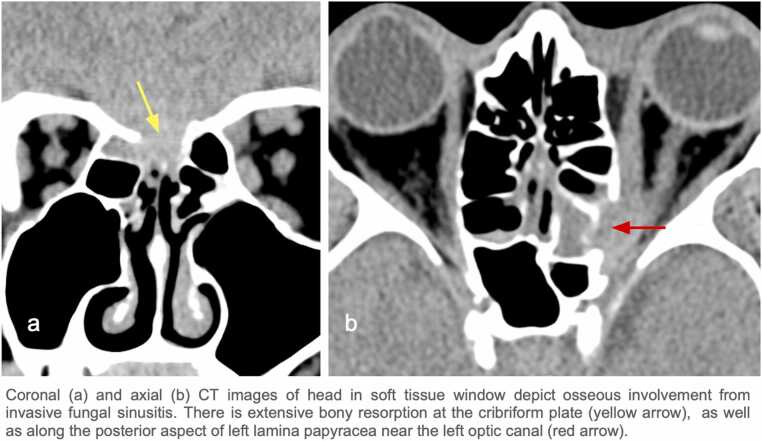
Coronal and axial CT images of head in soft tissue window depict osseous involvement from invasive fungal sinusitis.

Six months later, a repeat brain MRI showed substantial progression of CIFRS, including right frontal cerebral abscesses with increased vasogenic edema (Figure 1: B, Figure 2: B1). No new neurological deficits were present. The family decided to continue antifungal medication, including salvage micafungin infusion.

A year after initial presentation, the patient was readmitted for new-onset seizures. MRI demonstrated progression of vasogenic edema with evidence of mass effect in bilateral frontal lobes, an increase of cribriform plate mass, and encasement of the left optic nerve (Figure 1: C, Figure 2: C1). Vision in the patient’s right eye was fluctuating, ranging from slight decreases in acuity to complete blindness. It was decided to continue the current level of care to extend lifespan while balancing patient comfort.

Despite substantial family support and compliance with medication and appointments, the patient’s disease has continued to progress. This case therefore aims to inform on the morbid outcome of *Aspergillus* CIFRS even in the ideal patient when diagnosed in the advanced stage on initial encounter, further highlighting the importance of early evaluation and multidisciplinary intervention.

## Ethical approval

Our institution does not require ethical approval for reporting individual case reports. Written informed consent was obtained from the patient for publication of this case report and any accompanying images.

## CRediT authorship contribution statement

**Nikita Rajbhandari:** Validation, Resources, Data curation, Conceptualization. **Reed Jaworski:** Writing – review & editing, Validation. **Xiangyu Xie:** Writing – review & editing, Writing – original draft, Validation, Supervision, Project administration, Conceptualization. **Kremsmayer Tobias Paul:** Writing – review & editing, Writing – original draft, Visualization, Validation, Project administration, Conceptualization.

## Declaration of Competing Interest

The authors declare that they have no known competing financial interests or personal relationships that could have appeared to influence the work reported in this paper.
